# Freeze-cast SiOC ceramics supporting the growth of industrially relevant microorganisms

**DOI:** 10.1371/journal.pone.0325311

**Published:** 2025-06-12

**Authors:** Katharina Rauchenwald, Roghayeh Shirvani, Tobias Edtmaier, Matthias Steiger, Thomas Konegger

**Affiliations:** 1 Institute of Chemical Technologies and Analytics, TU Wien, Vienna, Austria; 2 Doctoral College CO2Refinery, TU Wien, Vienna, Austria; 3 Institute of Chemical, Environmental and Bioscience Engineering, TU Wien, Vienna, Austria; Mechanical and Nuclear Engineering, UNITED ARAB EMIRATES

## Abstract

Investigating the compatibility of ceramic support materials with industrially relevant microorganisms is a key starting point towards utilizing innovative ceramic frameworks for microbial culture support. This study demonstrates the biocompatibility of macroporous, freeze-cast SiOC monoliths with yeast *Komagataella phaffii* and bacteria *Escherichia coli*. In a first step, cultivations were carried out in the presence of non-macroporous SiOC materials pyrolyzed at 700 °C or 900 °C, which were further compared to Al_2_O_3_ and SiO_2_ as conventional ceramic and glass reference materials. Additionally, SiOC ceramics impregnated with 3 wt.% Cu were evaluated regarding cytotoxic effects, since Cu is recognized for its antimicrobial properties. Both *E. coli* and *K. phaffii* showed no growth inhibition in the presence of SiOC, yielding specific growth rates of 0.46 ± 0.01 h^−1^ and 0.088 ± 0.002 h^−1^, respectively, showing overall biocompatibility with SiOC. While *E. coli* showed growth inhibition in the presence of Cu via prolonged lag-phases, *K. phaffii* was resistant to Cu-modified SiOC. In the next step, adsorption of cells to macroporous SiOC was investigated after cultivation by electron microscopy of fracture surfaces of freeze-cast SiOC, structured with *tert*-butyl alcohol templating directional channels with pore opening diameters around 45 μm. Prevalent biofilm formation was observed within the channel walls with clear evidence for growth of *K. phaffii* as cell agglomerates. The study features promising results for promotion of the growth of *E. coli* and *K. phaffii* on freeze-cast SiOC ceramics, providing a versatile catalyst carrier design.

## 1. Introduction

Biocatalytic processes, catalyzed by whole cells or cell-derived subunits, operate at high selectivity and in ambient conditions, optimized by nature itself. Biocatalysts can be modified and designed for efficient biotechnological use [1], but the processes are predominantly performed in homogeneous operation in batch reactors where separation and reusability of the biocatalyst are limited. Immobilization is presented as a crucial strategy for integrating enzymes and cells to overcome challenges in biocatalysis and enable process intensification [2,3].

Immobilization of microorganisms can enhance process stability, recycling, and process control [2,4]. Various methods for immobilization exist, based on physical, magnetic or chemical interaction of cells or enzymes with the support material [5]. Common methods include encapsulation of cells in hydrogels (e.g., alginate [4,6]) thermogels (e.g., agarose [7]) or synthetic polymers (e.g. polyvinylalcohol [8], urethane methacrylate [9]). Magnetic immobilization [10] and covalent bonding techniques, such as utilization of covalent organic frameworks [2] or amine-functionalized silane compounds bound to activated glass with glutaraldehyde as linker molecule to cells [11], are reported. While covalent bonding is common for enzyme immobilization, straight-forward cell adsorption can be sufficient for immobilization of whole cells. Cell-to-surface and cell-to-cell contacts can be formed until cells attached to the surface start cell division, forming microcolonies, extracellular matrices and layers of cells anchored in biofilms [12]. Biofilm formation, a natural form of cell immobilization, is resource-efficient and improves metabolic function compared to other immobilization methods.

Ceramic materials possess high thermal and chemical stability, provide mechanical support and are suitable for operation under harsh conditions, hence, ceramic catalyst carriers support a wide range of catalytic conversions [13]. To ensure the viability of immobilized cells on porous ceramics, (i) the ceramic support material needs to be biocompatible and non-cytotoxic and must not provide a source of nutrients for the microorganisms, (ii) the support’s surface roughness and hydrophilicity is required to be suitable for sufficient cell adsorption, and (iii) the support must exhibit suitable pore morphology to ensure accessibility for mobile cells and sufficient provision of nutrients from the medium and, in the case of aerobic microorganisms, also oxygen for cell growth. Hence, the pore morphology should be in a suitable size range, interconnected and fully open, which depends on the interplay between the ceramic shaping and the selected ceramic material.

Among various techniques to shape porous ceramics such as foaming, sacrificial porogens, replica templating, or additive manufacturing, freeze-casting is an innovative technique that utilizes solidified solvent for pore templating. It enables precise control over the macropore morphology and directionality by selecting a suitable structure-directing solvent and applying a controlled thermal gradient, respectively. The method allows for the tailoring of pore sizes within the lower macropore size range. An open data initiative by Scotti et al. [14] offers an interactive tool to explore the effects of freeze-casting parameters on the resulting material properties. The macropore widths controllable by freeze-casting are usually in the range of approximately 1-100 μm, which matches the size range of whole cells (*e.g.,* yeast ~ 4-6 μm, [15] bacteria ~ 1-5 μm, [16,17] red blood cells ~ 6-8 μm [18]). Freeze-casting is also widely studied for manufacturing of porous biomaterials [19], e.g. for bone replacement materials [20].

To ensure compatibility of the support materials with microorganisms, the selected ceramic material must be non-cytotoxic and shapeable using the chosen processing method. The flexibility in material optimization and shapeability that comes with the polymer-derived ceramics route is of interest for various biomedical applications [21]. Polymer-derived ceramics [22] are a specific class of ceramics obtained from preceramic polymers in a pyrolytic treatment, yielding ceramic materials such as polysiloxane-derived SiOC. The pyrolytic treatment employed for the polymer to ceramic conversion affects both compositional (e.g., carbon content) as well as nanostructural features (e.g., transient microporosity) in the final ceramics [23]. The properties of SiOCs can be controlled by modifying the preceramic polymers. Ca- and Mg-modification can tune SiOC in terms of bioactivity to promote hydroxyapatite formation [24]. P-modified SiOC coatings enhance biocompatibility [25], while Ag-modified SiOC coatings provide antimicrobial properties to prevent implant-related infections in biomedical application [26]. Furthermore, Cu-SiOC composite materials are reported to reduce adhesion and biofilm formation of *Escherichia coli* bacteria [27].

The polymer-derived ceramics route is, in addition to the freedom in preceramic polymer modification, well-known for its extensive shaping capabilities, particularly toward porous ceramics [28]. Furthermore, the freeze-casting method can be combined with the polymer-derived ceramics route, offering a versatile material platform to tailor macrostructural as well as compositional properties [29]. In a previous study [30], freeze-casting of polysiloxane-derived ceramics provided a suitable material concept to support the continuous flow operation in organic synthesis employing ionic liquids as catalytically active sites. Furthermore, freeze-cast SiOC materials [31] were reported by another group to be suitable to support microorganisms for novel microbial fuel cell designs, where the interaction of SiOCs with electroactive bacteria was thoroughly studied [32]. Canuto de Almeida e Silva et al. also demonstrated biofilm growth on SiOC-based ceramic surfaces using *Escherichia coli* and *Bacillus cereus* [27]. However, only a few studies have been conducted using *K. phaffii* for biofilm formation on biomaterials and biofilm-based biocatalysis [33,34]. Ding et al. [33] demonstrated yeast cell attachment and biofilm structure formed by multiple cells and extracellular matrix to cotton fiber treated with succinic anhydride.

Due to the low cost, profound knowledge on genome, growth conditions and rapid doubling time, *E. coli* is widely used for expression of proteins on an industrial scale [35]. Other important organisms used in biotechnology are yeast species like the budding yeast *Saccharomyces cerevisiae* or the methylotrophic yeast *Komagataella phaffii* (formerly known as *Pichia pastoris*) which is well known for its high protein secretion capability. Methylotrophic yeast can grow on methanol [36], and when green methanol is considered, mass balances, e.g., for yeast biomass production, can be closed solely based on renewable feedstocks [37]. There have been various research studies where yeast cell-surface display technology was used to explore the application of *K. phaffii* as whole-cell biocatalyst [38–46]. In a recent study, Inokuma et al. [47] showed the outstanding reusability of *K. phaffii* as a whole-cell biocatalyst performing a one-pot enzymatic conversion of sucrose into cellobiose by co-displaying cellobiose phosphorylase and sucrose phosphorylase in this yeast. These studies have already demonstrated the advantages of *K. phaffii* as a whole-cell biocatalyst in improving industrial bioprocesses by offering a balance between efficiency, cost, and environmental sustainability. However, to our knowledge, no studies have compared yeast and bacterial growth within the channel structures of freeze-cast macroporous SiOCs designed for optimal accessibility.

This work explores the biocompatibility and growth behavior of industrially relevant microorganisms in the presence of novel porous ceramics, employing an innovative material design. In a first step, possible cytotoxic effects triggered by the presence of polymer-derived SiOCs compared to conventional ceramic materials were of interest to investigate biocompabitility. Additionally, SiOCs were impregnated with Cu to produce SiOC-Cu reference specimens creating an antimicrobial environment [48]. In the next step, freeze-cast SiOC structures of suitable macropore morphology and hydrophilicity were investigated in terms of supporting the growth of *E. coli* and *K. phaffii,* aiming for future use of macroporous SiOC as carrier structures for whole cells.

## 2. Materials and methods

### 2.1. Preparation of freeze-cast SiOC and ceramic reference materials

A chemical modification established elsewhere [49,50] was adapted to functionalize polysilsesquioxane with photoactive methacrylate in an acid-catalyzed hydrolysis reaction. Master batches of 40 wt.% total polymer content were prepared by dissolving 15 g polymethylsilsesquioxane (Silres MK, Wacker AG) in 28 g of *tert*-butyl alcohol (TBA) at 40 °C. 5 g of 3-(trimethoxysilyl)-propyl methacrylate (TMSPM, Sigma Aldrich) were added and stirred for 1 h at room temperature. One drop of HCl (37%) diluted in 2 g TBA was added while stirring at 500 rpm. Stirring was continued at 300 rpm for 20 h to complete functionalization, yielding a photosensitive polysiloxane solution in TBA (PSO-TBA). The master batch was diluted 1:1 by mass with TBA to yield 20 wt.% solid content. Phenyl bis (2,4,6-trimethylbenzoyl) phosphine oxide (Genocure*BAPO, Rahn AG) was added at 1 wt.% per polymer content as photoinitiator, sensitive at 400–405 nm. The solutions were homogenized and degassed using a planetary mixer (Thinky ARE-250).

PSO-TBA was freeze-cast unidirectionally on a custom-made set-up in polymethylmethacrylate (PMMA) tubular molds with inner diameters of 7 mm. The cold platform was cooled at a rate of ‐5 K min^−1^ to ‐120 °C. The specimens were subsequently illuminated for 20 min at a wavelength of 405 nm at ‐20 °C, using a 6 W LED setup. The specimens were then demolded, placed upside-down, and illuminated for another 20 min, stored at ‐20 °C overnight, freeze-dried for 20 h at 1 mbar, and again for 4 h at <0.1 mbar. The green bodies were pyrolyzed for 1 h at 700 °C or 900 °C in flowing Ar (0.5 L h^−1^) at a heating rate of 1 K min^−1^. After cooling to room temperature at ‐2 K min^−1^, freeze-cast SiOCs TBA-SiOC700 and TBA-SiOC900, respectively, were obtained. The top and bottom faces of the resulting monoliths were cut off (around 1.5 mm each) to ensure fully open-porous structures.

For the preparation of non-porous (dense, D) SiOC specimens (D-SiOC700, D-SiOC900), TBA in the master batch was removed using a rotary evaporator (60 mbar, 55 °C), and 1 wt.% per remaining polymer content (determined by differential weighing) of BAPO was subsequently added. The mixture was mixed, degassed, poured in silicone molds with 10 mm inner diameter, illuminated for 40 min at room temperature, and subsequently pyrolyzed using the previously stated conditions used for porous SiOC materials.

Dense Al_2_O_3_ specimens were prepared by cold-isostatic pressing of CT3000 SG (Almatis GmbH, D50 = 0.4 μm) with 3 wt.% Hoechst wax C in silicone molds of 4 mm inner diameter, pressed with 320 MPa for 30 s. Sintering was carried out at 1600 °C for 2 h at a heating rate of 3 K min^−1^, including a 30 min debindering step at 500 °C, and cooling to room temperature at a cooling rate of 3 K min^−1^, yielding D-Al_2_O_3_. For D-SiO_2_, fused silica tubes were cut. To study cytotoxic effects, freeze-cast and non-macroporous SiOCs were impregnated with copper (Cu). Targeting total loadings of 5 wt% Cu, respective amounts of Cu(NO_3_)_2*_3H_2_O were dissolved in 5 mL EtOH, and the specimens (TBA-SiOC900 and D-SiOC900) were added and ultrasonically treated for 20 min to remove entrapped air. EtOH was removed on a heating plate and dried at 110 °C overnight. Cu oxides were reduced in flowing N_2_/H_2_ mixtures (6 L min^−1^ of N_2_:H_2_ = 5:1) at 450 °C for 4 h (heating rate of 10 K min^-1^), yielding TBA-SiOC900-Cu and D-SiOC900-Cu with final Cu loadings of around 3 wt.%.

### 2.2. Strains, media, and cultivation of microorganisms in the presence of ceramic materials

The biocompatibility of the ceramics (thermally sterilized overnight at 120 °C) as well as positive control groups without any ceramics added were investigated using *E. coli* and yeast *K. phaffii* in biological triplicates.

M9 media supplemented with ferrous sulfate [51] and 10 g L−1 glucose was used as a minimal media for *E. coli* cultivations. *E. coli BL21* was transferred from cryo-stock onto a LB (Luria-Bertani) agar and incubated overnight at 37 °C. The pre-cultures in 100 mL flasks with 20 mL sterile LB broth media were inoculated with a single colony from the plate and incubated overnight at 37 °C at 180 rpm in a rotary flask shaker (Infors Multitron Standard). Cells were harvested by centrifuge at 5000 g for 5 min at 4 °C, and the supernatant was discarded. The cell pellets were resuspended in 10 mL M9 media, and the optical density at 600 nm (OD600) was measured using a spectrophotometer (Cell Density Meter Model 40, Fisher Scientific). Three sterile specimens per specimen type were transferred to each sterile 100 mL flask. The main culture of bacteria was performed in 20 mL of M9-glucose media with an initial OD600 of 0.5 incubated at 37 °C and 180 rpm for 24 hours. The growth progress was determined by recording the OD600 every hour.

*Komagataella phaffii* (*P. pastoris*) *CBS7435* was used as a yeast strain in this study. The minimal cultivation media, M2 citrate buffer supplemented with trace salt solution PTM0, was prepared as described before [37]. A single yeast colony from a YPD agar plate (1% yeast extract, 2% peptone, 2% glucose, 1.5% bacteriological agar) was inoculated into a 100 mL shake flask containing 20 mL of YPD broth (1% yeast extract, 2% peptone, and 2% glucose) as a preculture and incubated overnight in a rotary flask shaker at 28 °C and 200 rpm (Infors Multitron Standard, Bottmingen-Basel). After harvesting at 5000 g for 5 min at 4 °C by centrifuge and discarding of the supernatant, cells were resuspended in 10 mL M2 media, and the OD600 of the combined pre-cultures was set to reach an initial OD600 of 1.5 for the main culture. 20 mL of M2 media was inoculated with the pre-culture and added to the sterile specimens in a sterile 100 mL flask. Methanol as a carbon source was added to each flask at the beginning and after 16.5, 24, and 40.5 h (Fig 2). The OD600 readings were taken every 2 h to monitor the cell growth.

**Figure 1:**
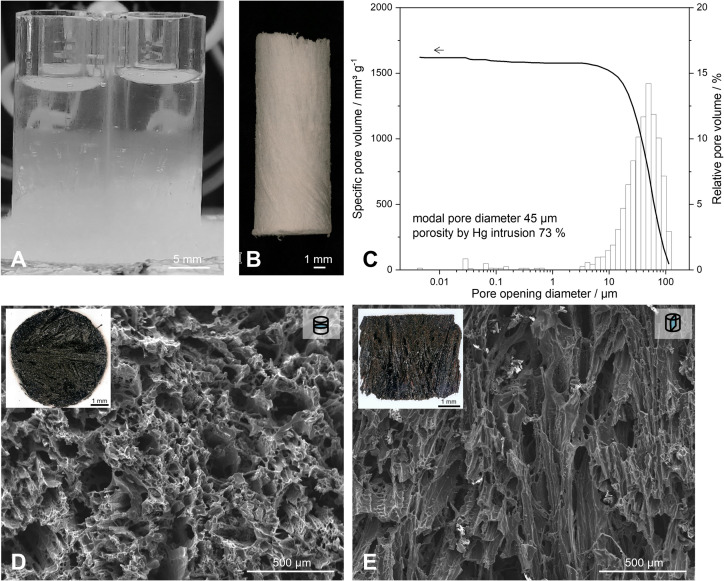
Macropore-tailoring of freeze-cast and pyrolyzed TBA-SiOC900 using *tert*-butyl alcohol as structure-directing solvent for templating directional porosity. (A) Freezing-front from solidified TBA in the polysiloxane solution at ‐56 °C platform temperature in the unidirectional freeze-casting set-up; (B) green body of polysiloxane obtained after sublimation of solidified TBA; (C) pore opening size distribution by mercury intrusion porosimetry of TBA-SiOC900 used for cultivation experiments; (D) cross-sectional SEM micrographs and (E) micrographs cut in axial direction (with freezing conducted in axial direction).

### 2.3. Characterization of ceramic specimens after cultivation by microscopy

The ceramic specimens were removed and washed in 10 mL of 15 vol.% glycerol/medium solution, and the specimens were stored at ‐80 °C in cryo-vials before freeze-drying for 24 h.

Investigation of the specimen morphology was conducted using digital optical microscopy (VHX-5000, Keyence) and a scanning electron microscopy (SEM, FEI Quanta 2050 FEG) at low excitation voltage (2.5–3 kV) and secondary electron detection. Porous specimens were fractured with a razorblade in the former freezing direction and sputtered with gold (AGAR Sputter Coater, 30 sec). Elemental information was derived by energy-dispersive X-Ray spectrometry (EDX, EDAX-AMETEK Octane Elite 55). Mercury intrusion porosimetry (Thermo Scientific Pascal 140/440) was used to determine pore opening size distributions.

## 3. Results and discussion

### 3.1. Freeze-casting of macroporous polysiloxane-derived SiOC ceramics

For macropore-templating of monolithic SiOC support structures, the goal was to obtain pore sizes >20 µm by freeze-casting to facilitate the intraporous growth of microorganisms. *Tert*-butyl alcohol was chosen as the structure-directing solvent during freeze-casting, templating prismatic porosity considered suitable for sufficient accessibility of the pore channels for *K. phaffii* and *E. coli* cells.

In Fig 1, a representation of the steps to obtain cellular SiOC monoliths from photopolymerization-assisted solidification templating, a variant of unidirectional freeze-casting with polymerization of polysiloxane by photopolymerization, is given. As shown in Fig 1A, a freezing front of solidified TBA propagates through the sample in the direction of the applied thermal gradient. After crosslinking and subsequent sublimation of solidified solvent, cylindrical porous green bodies are obtained (Fig 1B). After pyrolytic conversion at 900 °C, the polymer is converted to an amorphous ceramic exhibiting a broad albeit unimodal pore size distribution, with a mean pore opening diameter around 45 μm at a total intruded pore volume of 1.6 cm^3^ g^‐1^ (Fig 1C). Furthermore, freeze-casting using 20 wt.% polymer content in the preceramic polysiloxane and *tert*-butyl alcohol solution yielded an open porosity of 73 % in the final SiOC material. One SiOC monolith cut in parallel to the direction of former solidification is shown in Fig 1D and E, revealing the pore morphology, where pores are directional and aligned as a result of the unidirectional freeze-casting procedure.

Specimens are named by structure, pyrolysis temperature, and treatment. Non-macroporous SiOC is “D-SiOC700” (dense, 700 °C). Macroporous SiOC from tert-butyl alcohol is “TBA-SiOC900” (900 °C). Cu impregnation adds “-Cu” (e.g., “TBA-SiOC900-Cu”).

### 3.2. Biocompatibility of ceramic materials with the growth of microorganisms

In a first step, to investigate the biocompatibility of yeast (*K. phaffii*) or bacteria (*E. coli*) with ceramic materials, cultivations with no ceramic specimens added and in the presence of SiOC, SiO_2_, and Al_2_O_3_ specimens were carried out. Here, it was of interest whether a cytotoxic environment is imposed on the microorganisms by the presence of SiOC in reference to conventional ceramic materials. Furthermore, the effect of a particularly cytotoxic environment on the growth of the cells was studied by impregnating SiOCs with 3 wt.% Cu, since Cu is known to trigger cytotoxic effects and exhibit anti-microbial activity [48].

To determine the cell growth, the cultivation progress was monitored by measuring the optical density at a wavelength of 600 nm (OD600). Cultivations of both *E. coli* and *K. phaffii* were split into two batches each. Positive control groups were cultivated with no specimens added. All growth curves are represented as biological triplicates. Growth rates (μ) were calculated by least square fits during the exponential growth phase set at 2–6 h for *E. coli* and 0–24 h for *K. phaffii*. Bacteria grow significantly faster than yeast, with growth rates for *E. coli* in the range of 0.4–0.5 h^-1^ while *K. phaffii* grew with rates in the range of 0.08–0.09 h^-1^. In the literature, the maximal growth rate of *K. phaffii* is estimated at 0.11–0.12 h^-1^ in chemostat reactors on methanol [36]. The maximum growth rate for wild-type *E. coli* strains grown in stirred tank bioreactors, using M9 medium supplemented with 20 g L^-1^ of glucose was reported to be 0.67 h^−1^ [52]. The observed growth rates in non-idealized conditions can thus be considered reasonable.

**Figure 2:**
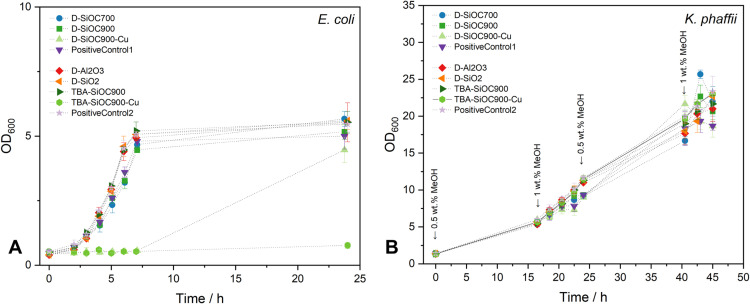
Growth curves for the cultivation of microorganisms in the presence of ceramic materials. Growth profile of (A) *E. coli* in M9 minimal media and (B) *K. phaffii* in M2 minimal media, with arrows marking the addition of MeOH. The error bars represent the standard deviation of independent biological triplicates.

After a short lag-phase, exponential growth was observed for *E. coli*, which entered a stationary phase after around 6–7 h (Fig 2A). SiOC and reference specimens, as well as the control groups, show similar growth rates in the exponential phase. In contrast, Cu-modified ceramics were shown to inhibit cell growth of *E. coli*. In the presence of D-SiOC900-Cu, the increase in the OD600 of *E. coli* was observed after about 24 h, indicating a prolonged lag-phase due to adaptation. In the presence of macroporous TBA-SiOC900-Cu, no significant growth of *E. coli* was observed in the timeframe investigated. The pyrolysis temperature during SiOC preparation, on the contrary, does not have an effect on the growth of *E. coli*, which shows comparable growth rates in the presence of D-SiOC700 (μ_D-SiOC700_*E. coli*_ = 0.38 ± 0.01 h^−1^) or D-SiOC900 (μ_D-SiOC900_*E. coli*_ = 0.38 ± 0.03 h^−1^) compared to the control (μ_PositiveControl1_*E. coli*_ = 0.38 ± 0.01 h^−1^). No prolonged lag-phase was observed in case of SiOC with non-modified surfaces, indicating no cytotoxic effect of SiOC and overall biocompatibility, as expected, considering that SiOC materials are widely studied for various biomedical applications [21]. Furthermore, considering a higher base growth rate of the second batch, there is no significant difference in the growth of *E. coli* in the presence of porous (μ_TBA-SiOC900_*E. coli*_ = 0.46 ± 0.01 h^−1^; μ_PositiveControl2_*E. coli*_ = 0.44 ± 0.04 h^−1^) or dense SiOC structures (μ_D-SiOC900_*E. coli*_ = 0.38 ± 0.03 h^−1^; μ_PositiveControl1_*E. coli*_ = 0.38 ± 0.01 h^−1^).

*K. phaffii* cultivations were carried out with methanol (MeOH) as the carbon source, which was added at times indicated by arrows given in Fig 2B. Unlike *E. coli*, growth of *K. phaffii* is not inhibited by Cu-modification with 3 wt.% Cu deposited on the surface of macroporous SiOCs. Considering the difference in the base growth rate of the two batches (μ_PositiveControl1_*K. phaffii*_ = 0.080 ± 0.003 h^−1^, μ_PositiveControl2_*K*. *phaffii*_ = 0.089 ± 0.002 h^−1^), neither pyrolysis temperature (μ_D−SiOC700_*K. phaffii*_ = 0.081 ± 0.001 h^−1^, μ_D−SiOC900_*K. phaffii*_ = 0.084 ± 0.003 h^−1^) nor Cu-modification (μ_TBA−SiOC900-Cu_*K. phaffii*_ = 0.089 ± 0.001 h^−1^, μ_TBA−SiOC900_*K. phaffii*_ = 0.088 ± 0.002 h^−1^) inhibited growth of *K. phaffii*. It can thus be concluded that SiOC does not impose cytotoxic and growth-inhibiting effects on *K. phaffii* while growing in minimal media and MeOH as the carbon source. Moreover, compared to *E. coli*, *K. phaffii* was resistant to the presence of Cu-modified SiOC. The contact-mediated killing of copper [53,54] was expected in the presence of Cu-modified specimens in both yeast and bacterial cultures [55–57]. However, it was shown that yeast cells generally exhibit higher resistance to copper than bacteria, mainly due to the yeast cell wall structure [58–60]. The result of the current study highlights the superiority of *K. phaffii* to cope with the presence of copper as a heavy metal. Indeed, Balakumaran et al. [54] showed improved copper-dependent recombinant protein production in *K. phaffii* while growth inhibition occurred at copper concentrations >10 mM. Another study by Chen et al. [58] also showed the ability of *K. phaffii* to be used as an environmentally friendly adsorbent for heavy metal ions such as Cu^+2^ from wastewater.

When comparing freeze-cast monoliths TBA-SiOC700 and TBA-SiOC900, penetration of water in the channels of macroporous monoliths was inhibited by a change in surface hydrophilicity imposed by a different degree of residual polymer functionalities. TBA-SiOC900 monoliths, which were obtained at a pyrolysis temperature where polymer-to-ceramic conversion is considered completed, could be fully immersed in water, thus providing sufficient interaction with aqueous media for cell penetration. In contrast, TBA-SiOC700 monoliths exhibited hydrophobic behavior and did not sink in water, which inhibited further investigations.

### 3.3. Adsorption of *E. coli* cells on freeze-cast SiOC ceramics

**Figure 3:**
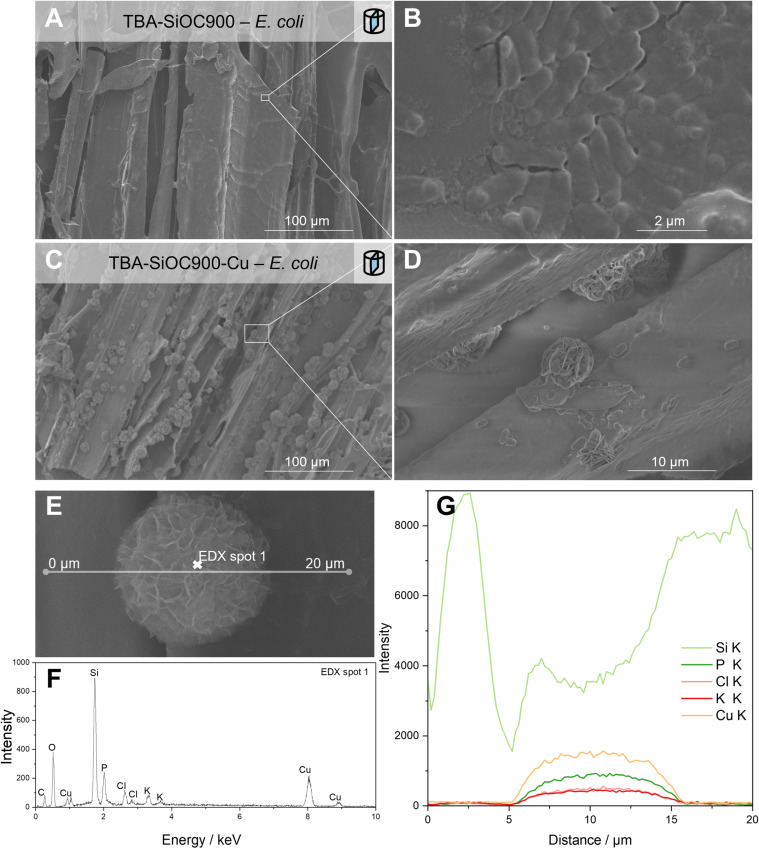
Cytotoxic effects of the presence of Cu on the adsorption of E. coli on freeze-cast SiOC. (A and B) Prevalently adsorbed, rod-shaped *E. coli* cells on the inner surface of TBA-SiOC900 after cultivation; (C and D) formation of “desert rose”-like structures on TBA-SiOC900-Cu after cultivation with *E. coli*; (E–G) accumulation of Cu species in the “desert rose”-like structures investigated by an elemental analysis line scan and (F) the EDX spectrum of the spot indicated in (E).

Consistent with lower growth rates of *E. coli* in the presence of Cu-impregnated specimens, a reduced cell adhesion tendency was observed. Occasionally, larger cell clusters were identified on non-porous D-SiOC900-*E. coli*. In contrast, on fracture surfaces of macroporous TBA-SiOC900-*E. coli*, cells sufficiently adsorbed on SiOC, as shown in Fig 3A and B. Furthermore, in the case of TBA-SiOC900-Cu-*E. coli*, ”desert rose”-like structures were found, as shown in Fig 3C and D. Since the phenomenon only appeared on macroporous and Cu-modified TBA-SiOC900-Cu-*E. coli* specimens after cultivation, it can be assumed that the formation of these structures can be attributed to an interaction of *E. coli* with Cu and/or associated oxides. EDX investigations, shown in Fig 3E–G, revealed an accumulation of Cu in these structures which were surrounded by cylindrically shaped *E. coli* cells.

Reports in the literature [21,24] describe hydroxyapatite formation leading to similar morphological occurrences on bioactive SiOC upon soaking in simulated body fluid, which contains comparable salt mixes [61] as the media used in this work. However, the increased concentration of Cu suggests another effect. Electroactive properties of *E. coli* can be correlated with a stabilization of micrometer sized Cu structures described elsewhere [62], which might lead to the observed “desert rose”-like structures. Detailed investigations on the interaction of *E. coli* and Cu-modified SiOC were out of the scope of this work. However, observations of decreased viability of *E. coli* bacteria in the presence of Cu-modified SiOCs and limited biofilm formation are in agreement with available literature [27].

### 3.4. Adsorption of *K. phaffii* cells on freeze-cast SiOC ceramics

**Figure 4:**
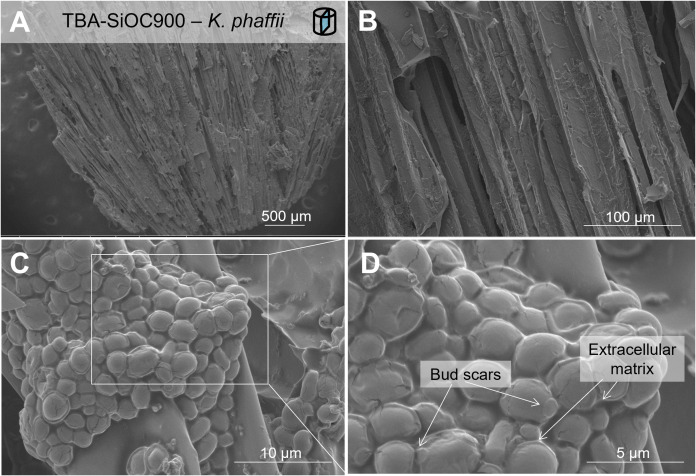
Biofilm of *K. phaffii* in the channels of a facture surface of freeze-cast SiOC. (A–C) SEM images in various magnifications; (D) indicators of growth of cells in cell agglomerates.

Yeast cell adhesion, in contrast, seemed to be similarly prevalent on both Cu- and non-modified SiOCs. A representative fracture surface is shown in Fig 4, revealing densely populated biofilms of *K. phaffii* on freeze-cast SiOC showing distinct indicators for growth regulated within the cell community:

(i) Rather heterogeneous cell size distributions were observed, with cell sizes around 1 - 4 μm, whereas *K. phaffii* cells are typically in a size range of 4–6 μm [17]. This could indicate that the cell cluster grew as an agglomerate. On the other hand, the budding mechanism of reproduction in yeast causes variations in cell size within the yeast population depending on the growth conditions and stage of the cell cycle [63].

(ii) Furthermore, a low number of budding scars are visible in the cell clusters, as indicated in Fig 4D, which are remnants of the reproduction process. It can be assumed that most are either covered by other cells or that cells did not completely separate after budding but grew as cell agglomerates.

(iii) The formation of an extracellular matrix, also indicated in Fig 4D, is another indicator for cell-cell adhesion leading to agglomeration, so-called flocculation [64], and subsequent biofilm growth of *K. phaffii* on freeze-cast SiOC.

It can thus be assumed from indicators (i–iii) that cell clusters formed from a few cells adsorbed on the SiOC surface and that new cells grown by budding were not desorbed into the media but ultimately formed biofilms.

In Fig 4D, also some cell ruptures are visible as a result of sample preparation, possibly stemming from the osmotic pressure during freeze-drying and/or from the electron beam in scanning electron microscopy.

Compared to biocompatibility-promoting SiOC coatings [24,25], freeze-cast SiOCs offer suitable properties for cell adhesion and biofilm growth without requiring further optimization through Mg- or P-modifications.

## 4. Conclusions

Freeze-casting of polysiloxane-derived ceramics provides a novel and versatile material platform to tailor essential properties such as wettability and pore structure, making the material concept promising for supporting biotechnological processes. This work shows that macroporous SiOC ceramic monoliths are suitable for supporting the growth of industrially relevant microorganisms such as *E. coli* and *K. phaffii*. No cytotoxic effects of SiOC materials pyrolyzed at different temperatures were observed. On the contrary, clear evidence of *K. phaffii* growth as cell agglomerates was observed, supported by electron microscopy images showing biofilm formation on the inner pore structures of SiOC.

Macroporous SiOC ceramics pyrolyzed at 900 °C provided sufficient wetting in aqueous media. The directional macroporosity with a broad, albeit unimodal size distribution averaging around 45 μm, which had been templated by solidified *tert*-butyl alcohol as structure-directing solvent in unidirectional freeze-casting, provided channel structures accessible for the whole cells. *K. phaffii* grew on freeze-cast SiOC ceramics as cell agglomerates, remaining on the surface after budding without being desorbed into the medium and forming biofilms. *K. phaffii* was further shown to be particularly resistant to cytotoxic environments, studied by impregnation of SiOCs with 3 wt.% Cu. In comparison, *E. coli* showed growth inhibition in the presence of Cu, indicated by prolonged lag-phases and generation of “desert rose”-like structures of accumulated Cu species.

In conclusion, freeze-cast SiOC ceramics fulfill biocompatibility and macropore morphology requirements to support microorganism growth. Freeze-casting of polymer-derived ceramics and the supported growth of whole cells in presence of the macroporous ceramics offers various tools for further development towards combining biotechnological applications with an innovative ceramic materials concept.
